# Denecimig (Mim8) prophylaxis in children with hemophilia A with or without factor VIII inhibitors

**DOI:** 10.1016/j.rpth.2026.106914

**Published:** 2026-08-20

**Authors:** Gili Kenet, Rubén Berrueco, Karin Fijnvandraat, Paula Kjöllerström, Matteo Luciani, Johnny Mahlangu, Nita Radhakrishnan, Runhui Wu, Andrea Páramo Florencio, Jay Jay Thaung Zaw, Manuel Carcao

**Affiliations:** 1 National Hemophilia Center and Institute of Thrombosis & Hemostasis, Sheba Medical Center, Tel-Hashomer, Israel; 2 Gray Faculty of Medicine, Tel Aviv University, Tel Aviv, Israel; 3 Pediatric Hematology Department, Hospital San Joan de Déu, Esplugues de Llobregat, Spain; 4 Department of Pediatric Hematology, Emma Children’s Hospital, Amsterdam UMC, University of Amsterdam, Amsterdam, Netherlands; 5 Pediatric Hematology Unit, Hospital Dona Estefânia, ULS São José, Lisbon, Portugal; 6 Onco-Hematology, Cell and Gene Therapy and Bone Marrow Transplant Clinic Area, Bambino Gesù Children’s Hospital, Rome, Italy; 7 Department of Molecular Medicine and Hematology, Faculty of Health Sciences, University of the Witwatersrand and NHLS, Johannesburg, South Africa; 8 Department of Pediatric Hematology Oncology, Post Graduate Institute of Child Health, Noida, India; 9 Hematology Oncology Center, Beijing Children’s Hospital, Capital Medical University, National Children's Health Center, Beijing, People's Republic of China; 10 Novo Nordisk A/S, Søborg, Denmark; 11 Division of Hematology/Oncology, The Hospital for Sick Children, University of Toronto, Toronto, Canada

**Keywords:** antibodies, factor VIIIa, hemophilia A, patient-reported outcome measures, quality of life

## Abstract

**Background:**

Denecimig (Mim8) is a next-generation activated factor (F)VIII mimetic bispecific antibody developed for subcutaneous prophylaxis in patients with hemophilia A (HA).

**Objectives:**

This study evaluated 52-week outcomes with denecimig in children from the phase 3 FRONTIER3 study (NCT05306418).

**Methods:**

Children aged 1 to 11 years with HA with or without FVIII inhibitors, received subcutaneous denecimig once every week (QW) for 26 weeks (Part 1). In Part 2, caregivers chose to continue QW or switch to once-every-month (QM) dosing for 26 weeks. Primary end point was the number of treatment-emergent adverse events. Assessed secondary outcomes included the number of treated bleeds and subtypes, injection-site reactions, antidenecimig antibodies, and caregiver-reported outcomes.

**Results:**

All 70 enrolled participants completed Parts 1 and 2 of the study. During Part 2, 38 participants continued QW and 32 switched to QM. All treatment-emergent adverse events were mild/moderate, and none led to discontinuation. No thromboembolic events or clinical evidence of neutralizing antibodies were reported; injection-site reactions were infrequent (∼1% of injections). Estimated mean annualized bleeding rates for treated bleeds were 0.53 (Part 1, QW), 0.42 (Part 2, QW), 0.25 (Part 2, QM), and 0.50 (Parts 1 and 2, QW); 74% to 88% of participants had zero treated bleeds; no bleeds were reported in inhibitor-positive children with HA with inhibitors. All 10 baseline target joints resolved. Caregivers reported reduced treatment burden and improved physical functioning, and 98% of caregivers preferred denecimig to prior therapy.

**Conclusion:**

Over 52 weeks, denecimig prophylaxis was well tolerated with no safety concerns and provided robust bleed protection, reduced treatment burden, and improved overall health-related quality of life in children with HA.

## Essentials

• FRONTIER3 studied denecimig prophylaxis in children with hemophilia A (HA).
• 70 children with HA aged 1 to 11 years received denecimig prophylaxis for 52 weeks.
• Denecimig was well tolerated, with few injection-site reactions reported (∼1% of injections).
• Up to 88% of children experienced zero treated bleeds with denecimig prophylaxis.

## Graphical abstract

**Figure:**
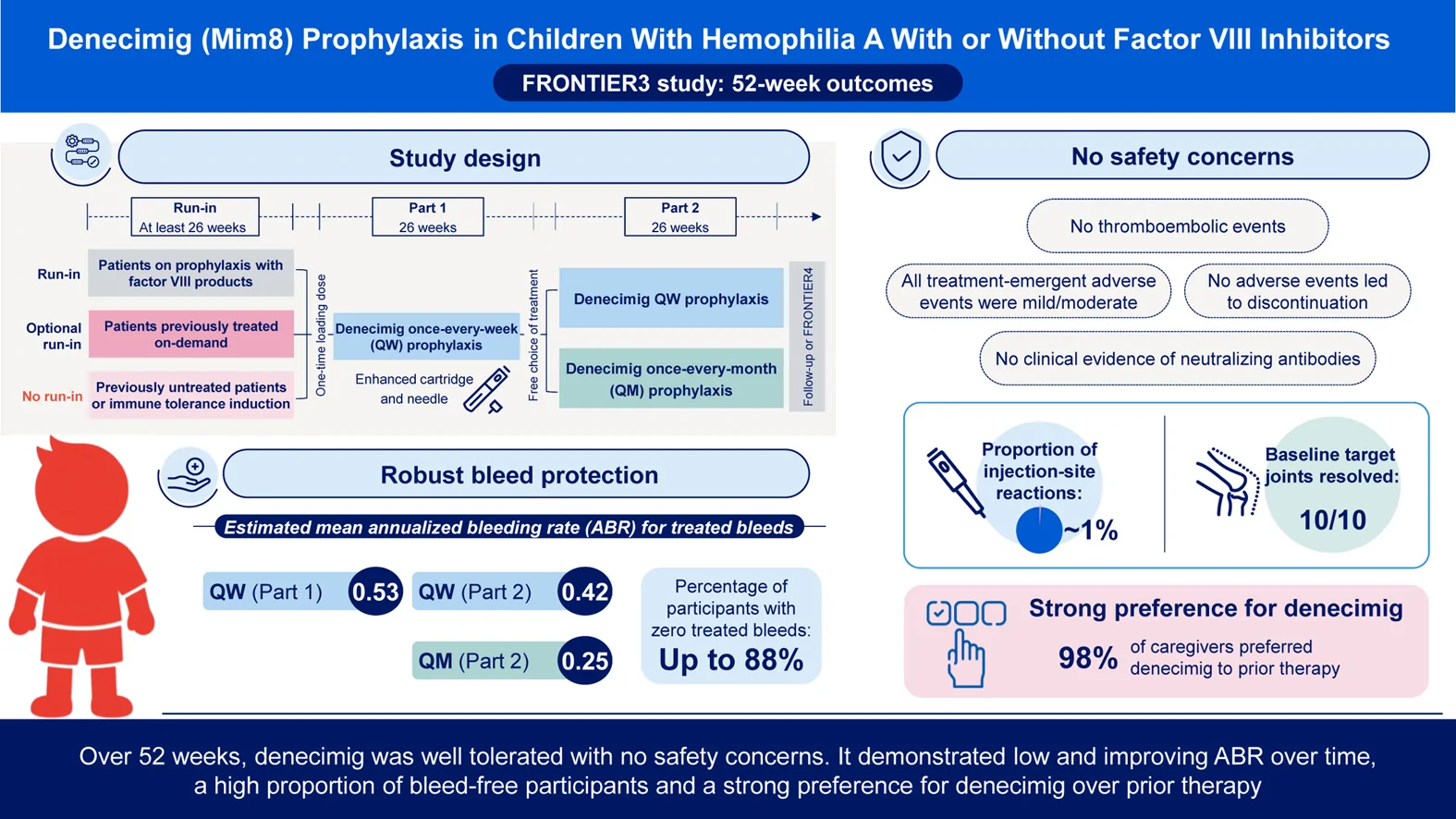

## 1 Introduction

Hemophilia A (HA) is a rare congenital bleeding disorder caused by a deficiency of coagulation factor (F)VIII [1]. Severe HA is associated with high morbidity due to spontaneous and trauma-induced bleeds, especially in muscles and joints, leading to chronic pain, irreversible joint damage, and hemophilic arthropathy [[1], [2], [3], [4], [5]]. In children, recurrent joint bleeds may impair bone development, restrict participation in physical activities, and negatively affect psychosocial development [[6], [7], [8], [9]]. Intracranial hemorrhage is a life-threatening complication that is more common in children, particularly neonates, in whom a recent systematic review estimated a pooled cumulative incidence of 2.1% (95% CI, 1.5-2.8) per 100 live births [10,11].

Although subcutaneous (s.c.) activated FVIII (FVIIIa) mimetics are now widely used for prophylaxis, intravenous FVIII clotting factor concentrate (CFC) replacement therapy remains necessary for the treatment of breakthrough bleeds, and continues to be used as primary prophylaxis in settings where nonfactor replacement is not available [1,12,13]. Regular intravenous infusions can be burdensome, particularly in children [1,14], and may require central venous access devices [15], which are associated with a variety of complications including infections, mechanical complications, and thrombosis [16,17]. Inhibitor development also remains a challenge in previously untreated patients (PUPs) [1,18]. These limitations underscore the continued need for innovative treatments in pediatric HA that reduce treatment burden and complexity, while maintaining effective bleed control.

FVIIIa mimetics offer an alternative prophylactic treatment option by providing stable, sustained hemostatic protection through less frequent dosing and enhanced efficacy in patients with or without FVIII inhibitors [19,20]. These bispecific antibodies mimic the function of endogenous FVIIIa by bridging activated FIX (FIXa) and FX to facilitate FX activation and restore thrombin-generation potential [21]. By reducing the frequency and complexity of treatment, FVIIIa mimetics aim to lessen the burden of care and improve quality of life for patients and their families [22,23]. Emicizumab was the first licensed nonfactor therapy and has transformed hemophilia care [24]. However, unmet needs remain. Both clinical and real-world data suggest that not all patients achieve complete bleed protection, with 63% and 70% of participants experiencing zero treated bleeds in clinical trials and real-world data studies, respectively [25]. A proportion of patients experience injection-site reactions (ISRs) or injection- related pain with emicizumab, as reported in both clinical trials and real-world data studies including pediatric, adolescent, and adult populations [[25], [26], [27]]. In addition, s.c. administration using vial and syringe systems involve weight-based dose calculations and multiple preparation steps, including needle attachment and transfer procedures, which can contribute to treatment complexity and potential drug wastage [28,29].

Denecimig (Mim8) is a next-generation, FVIIIa mimetic, fully human bispecific IgG4 antibody for s.c. prophylaxis in development for patients with HA with or without FVIII inhibitors [21]. Denecimig has been optimized to enhance FIXa cofactor activity, more closely replicating the function of natural FVIIIa [21]. In preclinical studies, denecimig demonstrated ∼15-fold higher thrombin-generation potency compared with emicizumab *ex vivo* and with a sequence-identical analog of emicizumab *in vitro* [21,30]. This enhanced potency enables denecimig to trigger thrombin generation at low concentrations, allowing a low 0.8 mL injection volume across all ages and dosing regimens [31]. Denecimig uses a novel tiered dosing approach, with a single loading dose followed by fixed doses determined by body weight range and chosen dosing frequency (once every week [QW] or once every month [QM]) [31]. Delivered via a prefilled pen device with a uniform injection volume using a 29-gauge needle, this strategy is designed to simplify administration and minimize the risk of dosing errors and drug wastage [31,32]. In the pivotal FRONTIER2 phase 3 study (NCT05053139) conducted in adults and adolescents with HA with or without inhibitors, denecimig prophylaxis significantly reduced treated bleeds when administered QW or QM, compared with on-demand treatment and previous CFC prophylaxis, and was well tolerated [33]. The present analysis reports 52-week safety, efficacy, pharmacokinetic, and caregiver-reported outcomes from FRONTIER3, the first phase 3 study of denecimig in pediatric patients (aged 1 to 11 years) with HA with or without FVIII inhibitors.

## 2 Methods

### 2.1 Study design

FRONTIER3 (NCT05306418) was a multinational, multicenter, open-label, single-arm phase 3 study evaluating the safety and efficacy of s.c. denecimig prophylaxis in children aged 1 to 11 years with HA with or without FVIII inhibitors (Figure 1). Participants who had previously received prophylaxis with FVIII or bypassing agents (previously treated patients [PTPs]) completed a mandatory 26-week run-in period to establish baseline bleeding rates, except for Chinese participants, for whom local regulatory requirements did not mandate a run-in period. Patients were considered to be on prophylaxis if they received bleeding prophylaxis at least twice weekly with a standard half-life FVIII or bypassing agent or once weekly with an extended half-life FVIII product, in accordance with the study protocol. For participants previously treated on-demand, the run-in period was optional, and these participants were required to have been prescribed FVIII concentrate or a bypassing agent for the treatment of bleeding episodes within the 26 weeks before screening; no run-in period was required for PUPs or those on immune tolerance induction (ITI).

**Figure 1:**
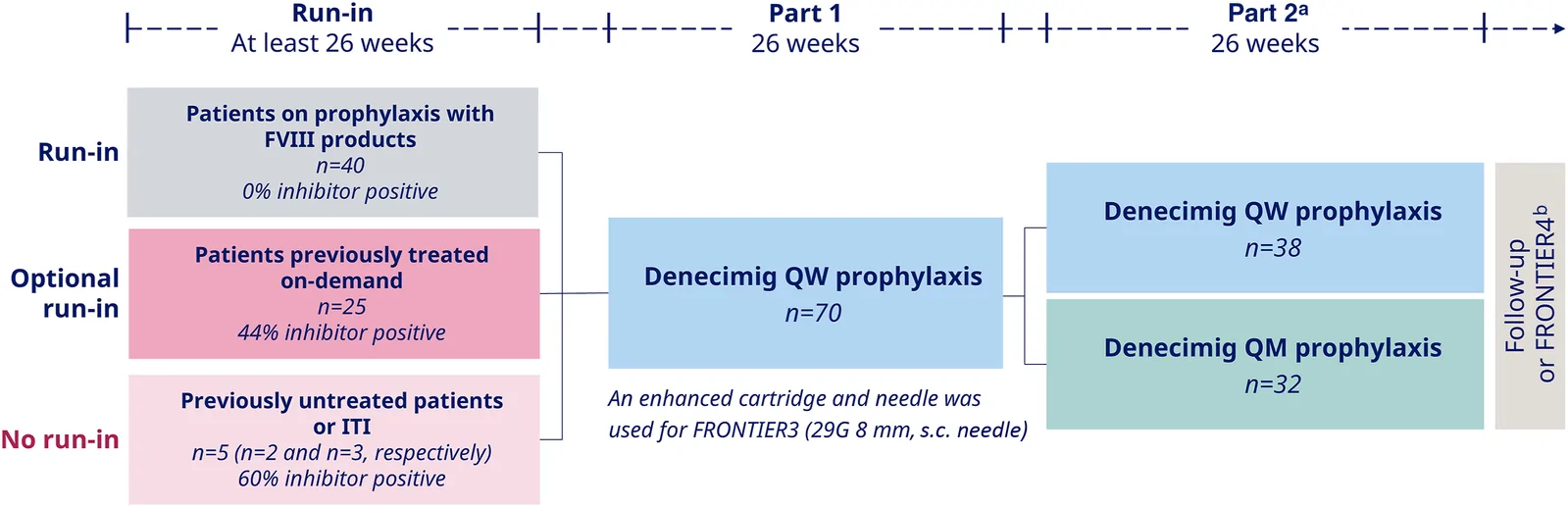
FRONTIER3 study design. ^a^For treatment in Part 2, caregivers and patients could choose to remain on denecimig QW or switch to denecimig QM. ^b^FRONTIER4 open-label extension (NCT05685238). F, factor; G, gauge; ITI, immune tolerance induction; QM, once every month; QW, once every week; s.c., subcutaneous.

During Part 1, participants received a one-time loading dose, followed by denecimig maintenance doses QW for 26 weeks. At the start of Part 2, caregivers and patients were given the choice to either continue denecimig QW or switch to QM dosing. The study aimed to achieve an approximately equal distribution between regimens and age groups, with at least 24 participants per regimen, including at least 12 participants aged 1 to 5 years and 12 participants aged 6 to 11 years. For those switching to QM dosing, the first QM dose replaced the next scheduled QW dose, after which QM dosing continued for the remainder of the study. After completing the 52-week treatment period, all participants were offered entry into the open-label extension study (FRONTIER4; NCT05685238); those who did not enroll were followed up for 21 weeks after their last denecimig dose.

### 2.2 Participants

Male or female children (aged 1 to 11 years) with congenital HA of any severity, with or without FVIII inhibitors, were eligible for inclusion in FRONTIER3. All PTPs were required to have been prescribed FVIII concentrate or a bypassing agent within the last 26 weeks before screening; any PTPs with endogenous FVIII activity of ≥1% must have experienced at least 1 treated bleed during this period. For PUPs, a diagnosis of severe HA (endogenous FVIII activity of <1%) was required. Patients undergoing ITI were eligible to enroll, provided ITI was not planned to continue after initiation of denecimig treatment.

Key exclusion criteria included prior use of nonfactor hemostatic prophylaxis within 6 months of enrolment for patients not undergoing a run-in period; the presence of other congenital or acquired coagulation disorders; and any history of thromboembolic disease requiring ongoing anticoagulation, apart from resolved catheter-associated thrombosis. Additional reasons for exclusion included other medical conditions that might increase the risk of bleeding or thrombosis or compromise adherence to the protocol, major surgery planned after screening, and initiation of ITI after the start of denecimig treatment.

### 2.3 Study intervention

All participants were dosed according to a tiered dosing regimen in which denecimig was administered QW or QM subcutaneously using an enhanced cartridge and needle with a fixed 0.8 mL administration volume (Supplementary Table 1). A one-time loading dose was given depending on body weight (9, 24, or 55 mg for patients with body weight <15, 15 to <45, and ≥45 kg, respectively). Maintenance dosing was dependent on body weight and dosing frequency: QW doses were 1.6, 4, and 9 mg, while QM doses were 9, 20, and 46 mg for patients weighing <15, 15 to <45, and ≥45 kg, respectively.

During denecimig prophylaxis, breakthrough bleeds were managed according to protocol guidance. For participants without FVIII inhibitors, bleeds were treated with FVIII concentrates (standard or extended half-life). For participants with FVIII inhibitors, recombinant activated factor VII was the preferred treatment; activated prothrombin complex concentrate could be considered for severe bleeds in which hemostatic control could not be achieved with recombinant activated factor VII or when no other therapeutic options were available, and only in consultation with the investigator, in line with protocol guidance. All rescue treatments were administered in accordance with approved prescribing information and protocol guidance, with dosing determined by the investigator.

### 2.4 Study end points

The primary objective was to investigate the safety of denecimig prophylaxis in children with HA with or without FVIII inhibitors. The primary end point was the number of treatment-emergent adverse events (TEAEs) from treatment initiation through week 52. Secondary end points comprised number of treated bleeds (including any spontaneous, traumatic, joint, and target joint bleeds treated with hemostatic medication), consumption of factor products per bleed, development of antidenecimig antibodies, number of ISRs, denecimig plasma concentration, and caregiver-reported outcomes. The presence of antidenecimig antibodies was monitored using tiered binding-antibody assays (Supplementary Methods) and evaluated for neutralizing capacity using pharmacokinetic and pharmacodynamic parameters. Antidenecimig antibodies were considered clinically neutralizing if they were associated with a clinically significant increase in the number or severity of treated bleeding episodes during denecimig prophylaxis. Assays were validated according to international guidelines and recommendations [[34], [35], [36]]. ISRs were defined as local signs and symptoms occurring at the injection site and were recorded as adverse events requiring additional data collection. ISRs were identified based on participant-reported symptoms and investigator visual assessment, with signs and symptoms including, but not limited to, erythema, induration, pain, itching, edema, hematoma, and ecchymosis. All bleeding episodes, whether treated or untreated, were initially reported by caregivers, who recorded bleed occurrence (including start and stop time), location, cause, and treatment details in diaries; events were subsequently reviewed and adjudicated by investigators at study visits based on protocol-defined parameters, with verification and correction of entries as required. All other end points, including safety assessments, were recorded by study investigators. Caregivers completed all patient-reported outcome instruments, hereafter referred to as caregiver-reported outcomes. Treatment burden was assessed using the Child Hemo-TEM (Hemophilia Treatment Experience Measure; lower scores indicate reduced treatment burden) [37], and health-related quality of life was assessed using the PedsQL (Pediatric Quality of Life Inventory; higher total and domain scores indicate better physical functioning and quality of life) 4.0 Generic Core Scales (parent report for young children [aged 5-7 years] and parent report for children [aged 8-12 years]) with change in the physical functioning domain analyzed as a secondary end point [38]. For the Child Hemo-TEM and PedsQL, end points were defined as change from baseline (week 0) to end of treatment (week 52). Caregiver preference was assessed at week 26 using the Caregiver H-PPQ (Haemophilia Patient Preference Questionnaire). Denecimig plasma concentrations, antidenecimig antibodies, and laboratory safety parameters (including hematology, biochemistry, coagulation markers [such as D-dimer, prothrombin fragments 1 and 2, and fibrinogen], and FVIII inhibitors) were evaluated at prespecified study visits using validated central laboratory assays, with additional sampling permitted if clinically indicated.

### 2.5 Statistical analyses

Analyses were performed using SAS Enterprise Guide V8.3. No formal sample size or power calculations were performed. The planned sample size of 50 participants expected to complete this study, and an equal sample size of 25 participants for each age group, were based on European Medicines Agency guidelines on the clinical investigation of recombinant and human plasma-derived FVIII products [39]. Descriptive statistics were presented for each study end point. Estimated annualized bleeding rates (ABRs) were derived using a negative binomial regression model, with the logarithm of prophylaxis duration included as an offset. Age group and type of previous treatment were also considered as factors, and results were presented with two-sided 95% CIs. For the comparison of bleed rates during standard-of-care run-in prophylaxis versus denecimig QW prophylaxis, a similar negative binomial model was applied, incorporating within-subject repeated measurements with an unstructured covariance structure. Denecimig plasma trough concentrations at steady state were analyzed using a mixed model based on log-transformed values, with participant as a random effect and treatment frequency and visit as fixed effects, and back-transformed estimates with corresponding CIs reported. The full analysis set and safety analysis set were defined as all participants who received at least 1 dose of denecimig, and analyses were conducted according to the treatment regimen received. Safety data were summarized descriptively, including the number and percentage of participants with adverse events (AEs), the number of events, and exposure-adjusted event rates. TEAEs were defined as any AE occurring from the first dose of denecimig through to the end of the follow-up period. AEs of special interest (AESIs; thromboembolic events including thrombotic microangiopathies) and hypersensitivity reactions were monitored with additional data collection, and bleeds were categorized as spontaneous, traumatic, joint, or target joint according to prespecified criteria. Analyses were examined across age groups (1 to 5 and 6 to 11 years) and dosing regimens, where appropriate. All remaining secondary end points will be evaluated using descriptive statistics based on the full analysis set. Mean change with SD and median change were calculated between baseline and visit 13 (end of study).

### 2.6 Study information

The study was initiated in April 2022 and was conducted across 29 sites and 16 countries. The study was conducted in accordance with the protocol approved by the ethics committee at each participating site. Study execution followed the principles outlined in the Declaration of Helsinki [40] and the International Council for Harmonisation Good Clinical Practice Guidelines [41]. All participants, or their parents or legal guardians, provided written informed consent. The study protocol, informed consent form, and all relevant supporting documents were reviewed and approved by the appropriate institutional review boards or independent ethics committees at each participating center prior to study initiation.

## 3 Results

### 3.1 Study population

Overall, 70 of 71 enrolled participants were exposed to denecimig, all of whom completed both parts of the study (Supplementary Figure 1). All participants were boys, 36 (51%) were aged 1 to 5 years, 60 (86%) had severe HA, 14 (20%) had FVIII inhibitors, and 65 (93%) weighed <45 kg (Table 1; participant race demographics reported in Supplementary Table 2; and details of previous treatment are reported in Supplementary Table 3). At the end of Part 1, the caregivers of 38 participants chose to remain on denecimig QW for Part 2, and the remaining 32 participants switched to denecimig QM. In total, participants were exposed to denecimig QW for 54.6 years (mean treatment analysis period, 0.78 years per participant) and denecimig QM for 16.10 years (mean treatment analysis period, 0.5 years per participant).

**Table 1:** Demographics and baseline characteristics.

Characteristic	Aged 1-5 y				Aged 6-11 y				Overall total
	PPX	OD	ITI/PUP	Total	PPX	OD	ITI/PUP	Total	
No. of participants	18	15	3	36	22	10	2	34	70
Age at baseline (y)									
Mean (SD)	3.3 (1.1)	2.5 (1.6)	2.0 (1.7)	2.9 (1.4)	8.6 (2.1)	8.2 (1.5)	8.0 (2.8)	8.4 (1.9)	5.6 (3.3)
Weight range (kg)									
<15	1 (5.6)	8 (53.3)	2 (66.7)	11 (30.6)	0	0	0	0	11 (15.7)
15 to <45	17 (94.4)	7 (46.7)	1 (33.3)	25 (69.4)	19 (86.4)	8 (80.0)	2 (100)	29 (85.3)	54 (77.1)
≥45	0	0	0	0	3 (13.6)	2 (20.0)	0	5 (14.7)	5 (7.1)
Region									
Africa	0	4 (26.7)	0	4 (11.1)	1 (4.5)	2 (20.0)	0	3 (8.8)	7 (10.0)
Asia	4 (22.2)	8 (53.3)	0	12 (33.3)	9 (40.9)	7 (70.0)	2 (100)	18 (52.9)	30 (42.9)
Eastern Europe	7 (38.9)	1 (6.7)	0	8 (22.2)	1 (4.5)	0	0	1 (2.9)	9 (12.9)
Europe, Middle East, and North America	7 (38.9)	2 (13.3)	3 (100)	2 (33.3)	11 (50.0)	1 (10.0)	0	12 (35.3)	24 (34.3)
Inhibitor status									
Negative	18 (100)	9 (60.0)	2 (66.7)	29 (80.6)	22 (100)	5 (50.0)	0	27 (79.4)	56 (80.0)
Positive	0	6 (40.0)	1 (33.3)	7 (19.4)	0	5 (50.0)	2 (100)	7 (20.6)	14 (20.0)
Severity of hemophilia									
Mild	0	1 (6.7)	0	1 (2.8)	1 (4.5)	0	0	1 (2.9)	2 (2.9)
Moderate	0	3 (20.0)	0	3 (8.3)	3 (13.6)	2 (20.0)	0	5 (14.7)	8 (11.4)
Severe	18 (100)	11 (73.3)	3 (100)	32 (88.9)	18 (81.8)	8 (80.0)	2 (100)	28 (82.4)	60 (85.7)

Values are n (%) unless specified. Participants were enrolled across 4 regions: Asia (China, n = 10; India, n = 9; Japan, n = 1; South Korea, n = 8; Taiwan, n = 2); Europe, the Middle East, and North America (Canada, n = 2; Israel, n = 2; Italy, n = 2; Netherlands, n = 1; Portugal, n = 3; Spain, n = 3; Switzerland, n = 1; United Kingdom, n = 4; United States, n = 6); Eastern Europe (Poland, n = 9); and Africa (South Africa, n = 7). Inhibitor status was defined based on prescription of bypassing agents (factor [F]VII or activated prothrombin complex concentrate) within 6 months prior to screening, receipt of ITI, or at least 1 documented high-titer FVIII inhibitor result (≥5 Bethesda Units) up to week 26.

ITI, immune tolerance induction; OD, on-demand; PPX, prophylaxis; PUP, previously untreated patient.

Baseline demographics and disease characteristics were broadly similar between participants who remained on denecimig QW and those who switched to QM dosing in Part 2. Mean age was comparable between groups (5.7 years for QW vs 5.5 years for QM), with most participants across both groups within the 15 to <45 kg weight range. Among participants with FVIII inhibitors (n = 14), equal numbers remained on QW (n = 7) or switched to QM (n = 7). Distributions were also similar with respect to hemophilia severity, prior treatment regimen, and ITI use, and target joints at baseline (4 participants in each group).

### 3.2 Safety outcomes

A total of 231 TEAEs were reported in 58 (83%) participants across the 52-week treatment period (incidence rate, 3.27 per patient-year) (Table 2); 184 events were reported in 54 (77%) participants receiving denecimig QW (54.6 years of total exposure), and 47 events were reported in 20 (63%) participants receiving denecimig QM (16.10 years of total exposure). All AEs were classified as mild (n = 205 AEs in 57 participants) or moderate (n = 26 AEs in 13 participants) in severity. Most (n = 187 AEs in 56 participants) were assessed as unlikely to be related to denecimig; 44 AEs in 13 participants were assessed as probably or possibly related. A total of 7 serious AEs were reported by 5 participants, comprising 5 infections, 1 nonconcussive head injury in a 4-year-old patient following an accidental fall, and 1 suspected myositis investigated by ultrasound but unconfirmed (Supplementary Table 4); all serious AEs were recovered or resolved, and none were considered related to denecimig. There were no fatal AEs, no hypersensitivity reactions or AESIs observed, and no participants permanently discontinued denecimig.

**Table 2:** Summary of adverse events.

Characteristic	Denecimig QW (Parts 1 and 2)		Denecimig QM (Part 2)		Total	
	n (%)	E (R)	n (%)	E (R)	n (%)	E (R)
No. of participants	70	—	32	—	70	—
Total exposure time (y)	54.60	—	16.10	—	70.70	—
All AEs	54 (77.1)	184 (3.37)	20 (62.5)	47 (2.92)	58 (82.9)	231 (3.27)
Serious AEs	3 (4.3)	4 (0.07)	2 (6.3)	3 (0.19)	5 (7.1)	7 (0.10)
AESI (thromboembolic events)^a^	0 (0)	0 (0)	0 (0)	0 (0)	0 (0)	0 (0)
AEs by severity						
Mild	53 (75.7)	164 (3.00)	18 (56.3)	41 (2.55)	57 (81.4)	205 (2.90)
Moderate	10 (14.3)	20 (0.37)	4 (12.5)	6 (0.37)	13 (18.6)	26 (0.37)
Severe	0 (0)	0 (0)	0 (0)	0 (0)	0 (0)	0 (0)
Probably or possibly related to denecimig^b^	12 (17.1)	34 (0.62)	4 (12.5)	10 (0.62)	13 (18.6)	44 (0.62)
AEs leading to permanent discontinuation of trial product	0 (0)	0 (0)	0 (0)	0 (0)	0 (0)	0 (0)
AEs with additional data collection category						
Injection-site reactions^a^	7 (10.0)	26 (0.48)	1 (3.1)	5 (0.31)	7 (10.0)	31 (0.44)
Hypersensitivity reactions^b^	0 (0)	0 (0)	0 (0)	0 (0)	0 (0)	0 (0)
Medication error, misuse and abuse	6 (8.6)	14 (0.26)	—	—	6 (8.6)	14 (0.20)

AE, adverse event; AESI, adverse event of special interest; *E*, No. of adverse events; QM, once every month; QW, once every week; R, No. of adverse events per patient year of exposure (*E*/total exposure time in study).

a Investigator’s categorization following clinical study protocol.

b Investigator’s categorization with probable or possible causal relationship to trial product.

A total of 7 (10%) participants treated with denecimig reported ISRs, observed in 31 of 3079 (1.01%) injections. One participant alone reported 24 (77% of all) ISRs across the treatment period (19 in Part 1 while receiving denecimig QW and 5 in Part 2 while receiving denecimig QM). The most common signs/symptoms reported were erythema and induration, and 2 of the ISRs included pain or burning as a symptom. Most ISRs were transient, and all were mild in severity.

Treatment-emergent antidenecimig antibodies were reported in 5 (7.1%) participants, all of whom qualified as low titer (below the regulatory detection threshold of 100 ng/mL). No clinical evidence of neutralizing antidenecimig antibodies was observed, as judged by stable denecimig concentrations and activated partial thromboplastin time remaining within expected ranges and by the absence of any correlation with bleed outcomes or other immunogenicity concerns (Supplementary Figure 2).

No thromboembolic events occurred, and fluctuations in D-dimer or prothrombin fragments were not associated with any events of clinical relevance. There were no consistent derangements in coagulation or other laboratory parameters (Supplementary Figure 3), and no pattern was detected in relation to denecimig treatment or to the occurrence of bleeds.

### 3.3 Efficacy outcomes

There were 39 treated bleeds observed over the 52-week treatment period (35 in 20 participants receiving denecimig QW and 4 in 4 participants receiving denecimig QM), all of which were mild or moderate in severity. Most bleeds (31 [80%]) were traumatic, 6 (15%) were spontaneous, and 2 (5%) were surgery related. Nearly half (17 [44%]) of bleeds were joint bleeds, of which 4 were spontaneous and 13 were traumatic. A total of 6 (15%) skin bleeds (all traumatic), 6 (15%) mouth, gum, or nose bleeds (2 spontaneous, 2 traumatic, and 2 surgical), and 1 muscle bleed (traumatic) were observed. The remaining 9 bleeds were defined as ‘other’ in location, all of which were traumatic. Of the 222 total bleeds reported, 183 (82%) did not require treatment, as determined by the investigator.

The median individual ABR for participants treated with denecimig QW in Part 1 was 0.00 (IQR, 0.00-1.73). In Part 2, the median individual ABR was 0.00 (IQR, 0.00-0.00) for both denecimig regimens (QW and QM), and 0.00 (IQR, 0.00-1.00) for QW across the full treatment period (Parts 1 and 2).

In Part 1, the estimated mean ABR for treated bleeds in the 70 participants receiving denecimig QW was 0.53 (95% CI, 0.30-0.94) (Figure 2A), with 74% (n = 52) of participants experiencing zero treated bleeds over a mean treatment analysis period of 26 weeks (Figure 2B). As per the protocol, 36 participants previously receiving prophylaxis entered the standard-of-care run-in for a minimum of 26 weeks and were included in the analysis comparing bleed rates during standard-of-care run-in prophylaxis with denecimig QW prophylaxis. Of these participants, 22 (61%) experienced treated bleeds during the run-in: 51% (n = 30) were spontaneous and 47% (n = 28) traumatic, with 1 (2%) surgical bleed. During the run-in, the estimated mean ABR for treated bleeds was 2.73 (95% CI, 1.86-4.02), with a median individual ABR of 1.91 (IQR, 0.00-3.95). After switching to denecimig QW in Part 1, the estimated mean ABR in these participants was 0.94 (95% CI, 0.56-1.56), demonstrating a relative reduction in ABR of 65.7% (ABR ratio, 0.343; 95% CI, 0.198-0.594) (Figure 3).

**Figure 2:**
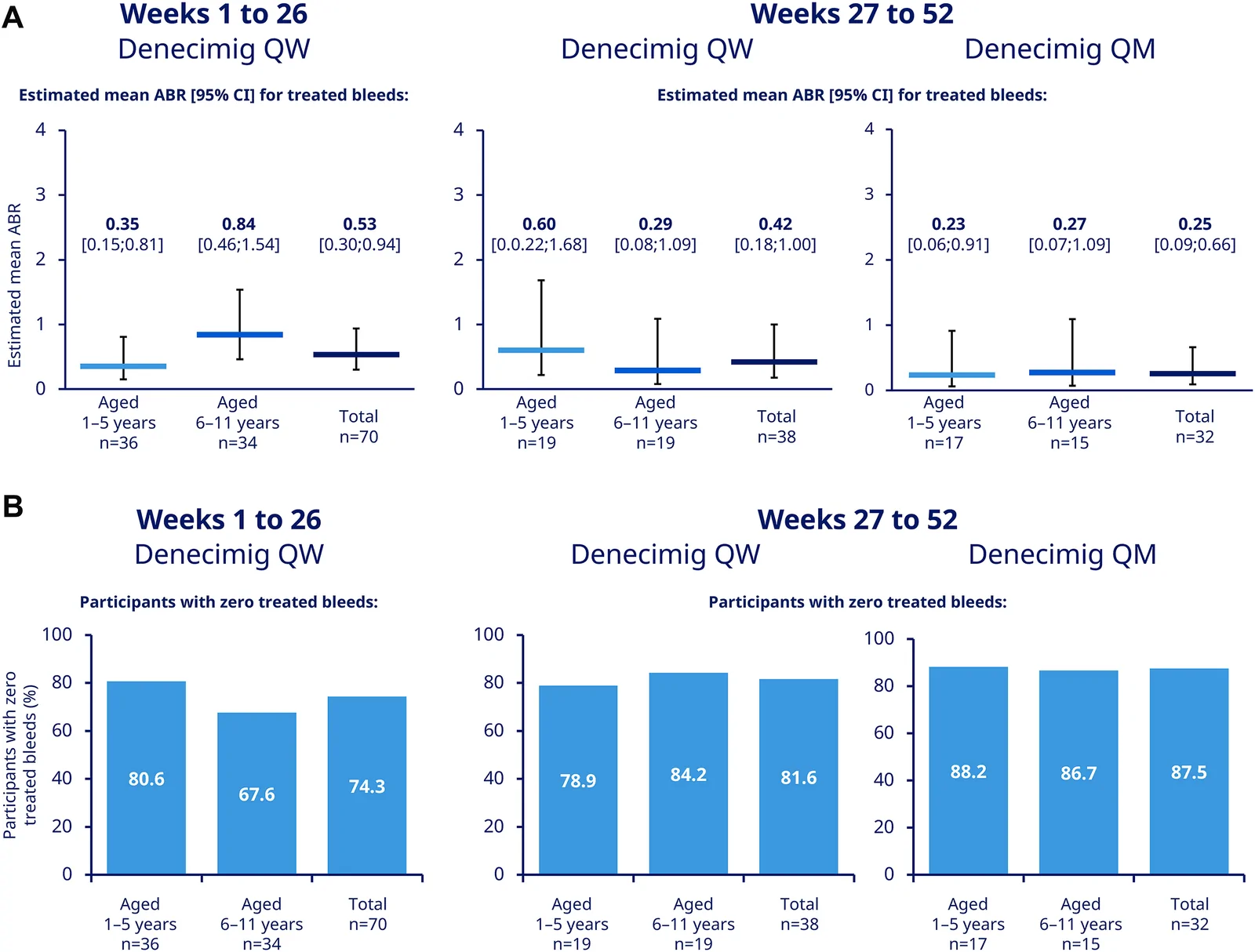
(A) Treated bleeds on denecimig QW and QM prophylaxis. (B) Proportion of participants with zero treated bleeds on denecimig QW and QM prophylaxis. Error bars indicate 95% CI. The negative binomial model used for ABR assessment includes age group and previous treatment as factors, and treatment duration is used as an offset. ABR, annualized bleeding rate; QM, once every month; QW, once every week.

**Figure 3:**
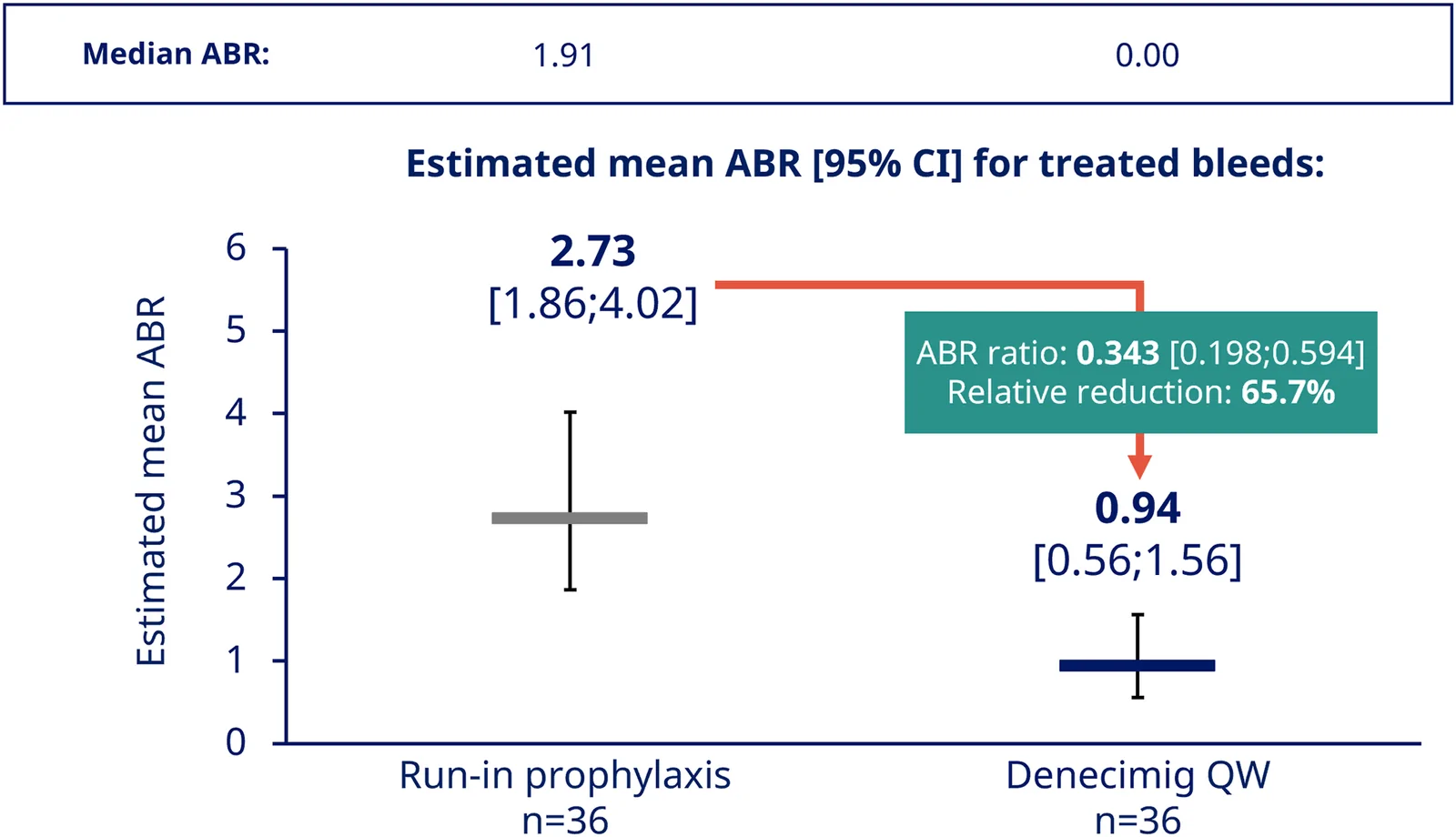
ABR while receiving denecimig QW prophylaxis vs previous standard-of-care prophylaxis treatment during the run-in. Error bars indicate 95% CI. The analysis includes only prophylaxis participants who completed the mandatory 26-week run-in period (n = 36) and follows them while they are on denecimig QW during Part 1. The negative binomial model includes the logarithm of prophylaxis duration as offset, age group as factor, and within-subject repeated measurements using an unstructured covariance structure. ABR, annualized bleeding rate; QW, once every week.

In Part 2, the estimated mean ABR for treated bleeds was 0.42 (95% CI, 0.18-1.00) in the 38 participants who remained on denecimig QW (Figure 2A), of which 82% (n = 31) reported no treated bleeds (Figure 2B). The estimated mean ABR in the 32 participants who switched to denecimig QM was 0.25 (95% CI, 0.09-0.66) (Figure 2A), of whom 88% (n = 28) reported no treated bleeds over a mean treatment analysis period of 26 weeks for both groups (Figure 2B). For all participants receiving denecimig QW across Parts 1 and 2 (all 70 participants in Part 1 and 38 participants who continued denecimig QW in Part 2), the estimated mean ABR was 0.50 (95% CI, 0.30-0.85) over a mean treatment analysis period of 41 weeks. Among participants with FVIII inhibitors, 100% (n = 14) had zero treated bleeds across both parts 1 and 2. During Part 1, the 31 participants who subsequently switched to QM dosing had an estimated mean ABR of 0.45 (95% CI, 0.18-1.13) for treated bleeds over the full Part 1 period, with 77% (n = 24) experiencing zero treated bleeds; 1 participant had not yet made their dosing decision before beginning Part 2.

Most bleeds were treated with 1 to 2 injections of FVIII (including standard recombinant CFC, extended half-life factor concentrate or plasma-derived FVIII): 22 (88%) bleeds in Part 1, 10 (100%) bleeds in Part 2 QW, and 4 (100%) bleeds in Part 2 QM. At baseline, target joints were present in 8 (11%) participants previously treated on-demand, comprising 1 participant aged 1 to 5 years (n = 1 target joint) and 7 participants aged 6 to 11 years (n = 9 target joints); all 10 target joints were resolved by the end of the treatment period.

### 3.4 Denecimig plasma concentration

Observed denecimig plasma concentrations were stable throughout the study, with little variation in exposure (Figure 4).

**Figure 4:**
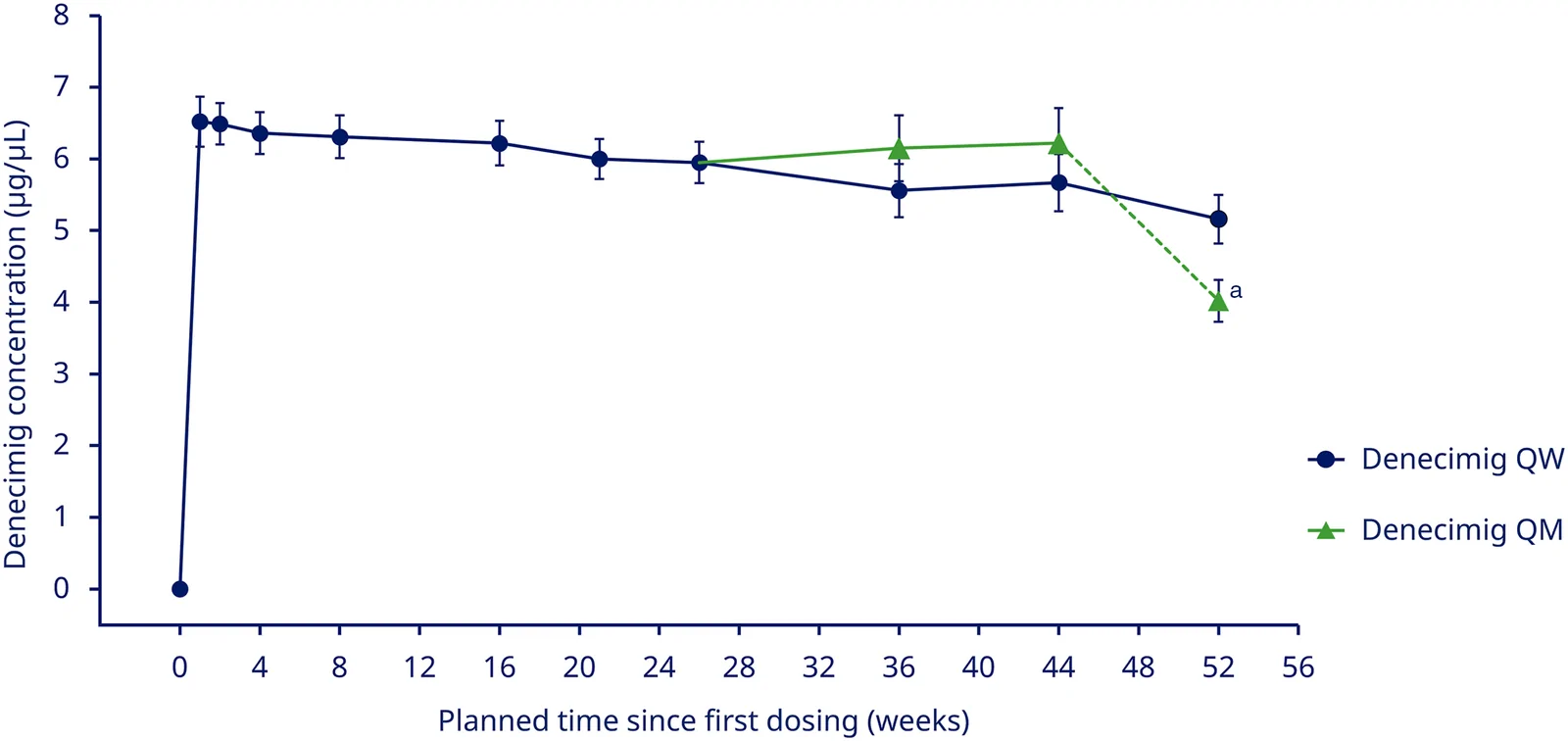
Mean denecimig plasma concentration. ^a^Most denecimig QM samples at weeks 36 and 44 were collected approximately 2 weeks after the last dose (steady state), whereas QM samples at week 52 were collected approximately 4 weeks after the last dose (predose trough), in accordance with the protocol-defined visit schedule. The lower mean concentration observed at week 52 therefore reflects the timing of sample collection rather than a reduction in denecimig exposure. The mean body weight for participants in FRONTIER3 increased during the study. Error bars indicate standard error of the mean. QM, once every month; QW, once every week.

### 3.5 Caregiver-reported outcomes

Treatment burden of administrating the prophylactic treatment was reduced over the 52-week study period as evaluated by the Child Hemo-TEM. Mean (SD) total scores decreased by −17.3 (22.6) points overall, −18.3 (26.8) points in the denecimig QW group, and −15.8 (16.1) points in the denecimig QM group, reflecting a reduction in caregiver-perceived treatment burden (Figure 5A). Physical functioning improved over the 52 weeks, with a mean (SD) increase in PedsQL physical functioning domain score of +10.4 (22.6) points overall, +18.0 (19.2) points in the QW group, and +1.1 (23.5) points in the QM group, compared with baseline levels, indicating improvements in patient mobility, strength, and ability to perform daily tasks (Figure 5B). The lower QM mean was driven by a single outlier; excluding this patient, changes were comparable across dosing groups. Other aspects of quality of life also improved at week 52, with mean (SD) overall increases of: +13.9 (16.3) points in emotional functioning (+16.2 [14.0] points QW; +11.1 [18.8] points QM), +15.6 (21.7) points in social functioning (+21.5 [17.8] points QW; +8.6 [24.4] points QM), and +16.6 (24.2) points in school functioning (+23.8 [19.4] points QW; +7.9 [27.2] points QM). Quality of life overall also improved at week 52, with a mean (SD) increase of +13.6 (18.4) points overall, +19.6 (14.7) points in the QW group, and +6.4 (20.3) points in the QM group (Supplementary Figure 4) as assessed by the PedsQL total score. To contextualize the magnitude of observed changes in health-related quality of life, a post hoc analysis using the standard error of measurement (SEm) was conducted for PedsQL change scores between baseline and the end of Part 2. In the post hoc SEm analysis, 68% of participants demonstrated an improvement in PedsQL total score that was greater than or equal to the SEm threshold (4.59). Across individual PedsQL domains, 52% to 71% of participants exceeded the corresponding SEm threshold (7.04-10.41 across the domain scores) (Supplementary Table 5). Among caregivers who completed the Caregiver H-PPQ at week 26 (n = 60), 98% (n = 59) expressed a preference for denecimig over their child’s previous treatment, with 73% (n = 44) of caregivers reporting a very strong preference for denecimig.

**Figure 5:**
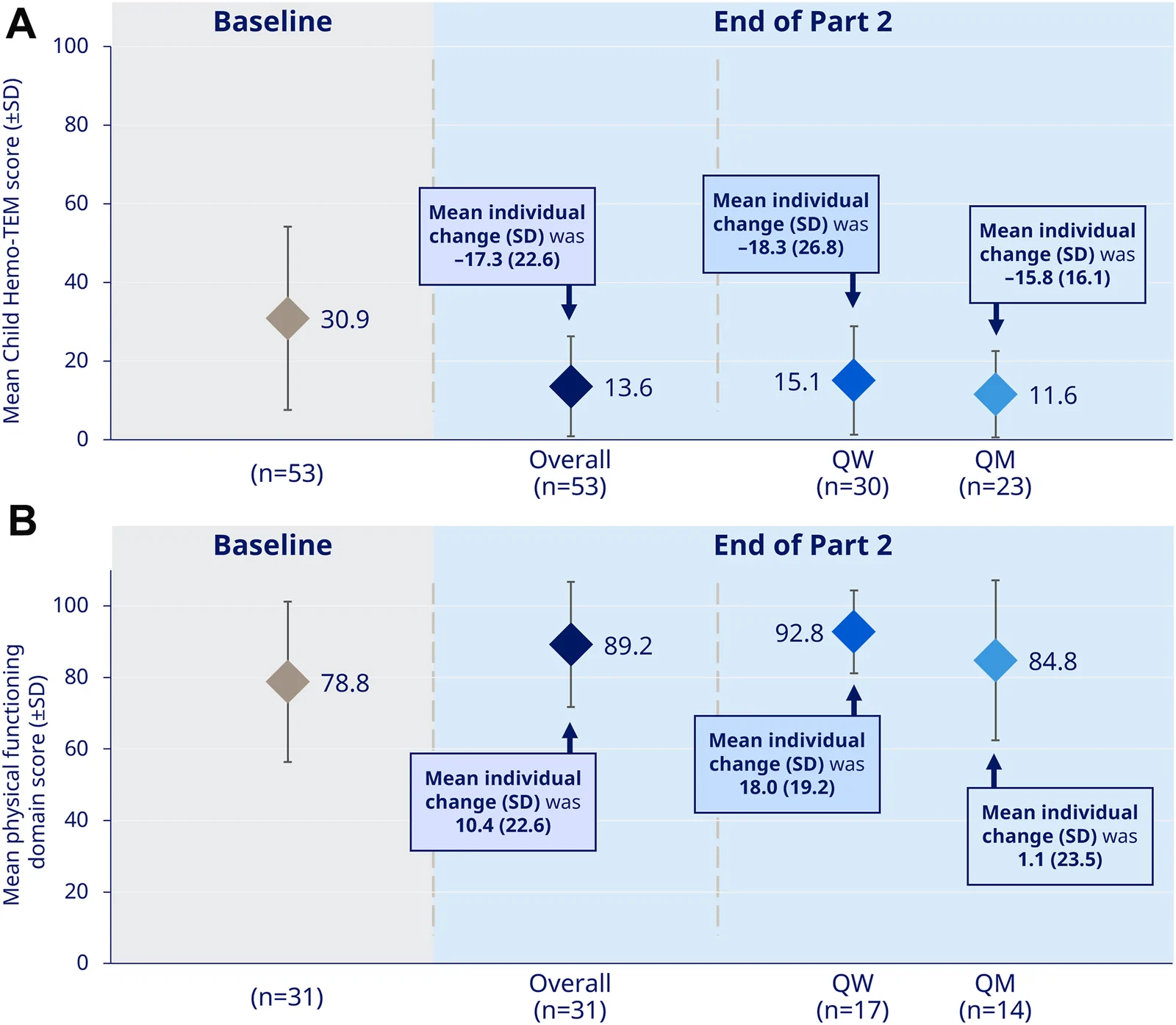
(A) Change in Child Hemo-TEM total score with denecimig prophylaxis from baseline to week 52. (B) Change in PedsQL physical functioning domain scores with denecimig prophylaxis from baseline to week 52 in participants aged ≥5 years. Error bars indicate SD. Gray diamond indicate baseline domain scores. Blue diamonds represent end of Part 2 mean physical functioning domain scores for the overall population, QW, and QM dosing groups. (A) For Child Hemo-TEM, scores range from 0 to 100; a lower score indicates less treatment burden. (B) For PedsQL, scores range from 0 to 100 for both total and domain scores; a higher score indicates better physical functioning. Results from the PedsQL questionnaires are obtained from the Parent Report for Children (aged 8 to 12 years), version 4.0. and the Parent Report for Young Children (aged 5 to 7 years), version 4.0. Data from these two versions were pooled. For both questionnaires, mean change and SD include participants who reached the end of Part 2 (week 52) and who had responded to the questionnaire at baseline and week 52. Hemo-TEM, Hemophilia Treatment Experience Measure; PedsQL, Pediatric Quality of Life Inventory; QM, once every month; QW, once every week.

## 4 Discussion

During the 52-week FRONTIER3 study, denecimig QW and QM prophylaxis was well tolerated, with no thromboembolic events, neutralizing antibodies or other safety concerns observed in children with HA, with or without FVIII inhibitors. Denecimig demonstrated consistent bleed protection, whereby the majority of participants on both dosing frequencies experienced zero treated bleeds and all baseline target joints resolved by the end of the treatment period. Caregivers reported markedly reduced treatment burden, improvements in physical functioning, and preference of denecimig over prior therapies. Collectively, these findings highlight the potential of denecimig as an FVIIIa mimetic capable of addressing key unmet needs in pediatric patients with HA.

Denecimig was well tolerated, with no participants discontinuing treatment, no hypersensitivity reactions, and ISRs reported in only 7 (10%) participants, corresponding to ∼1% of all injections. This low incidence of ISRs may be attributable to the small injection volume, the combination of excipients and 29 gauge needle used [[42], [43], [44]]. *In vitro* thrombin-generation analyses have shown that denecimig does not show an additive effect to FVIII at standard doses and supports the concomitant use of FVIII or FVIIa for breakthrough bleed management, whereas concomitant use of activated prothrombin complex concentrate requires caution because of the potential for excessive thrombin generation and thrombotic complications [45]. Safety outcomes in pediatric patients are consistent with those reported in the denecimig phase 3 FRONTIER2 study of adolescents and adults [33].

Denecimig prophylaxis provided robust bleed protection across both dosing frequencies, with observed ABRs comparable with those reported in FRONTIER2 [33]. A slight decline in denecimig plasma concentrations over time was noted; however, this modest variability in exposure is likely largely attributable to growth-related increases in body weight in this pediatric population. A more noticeable decrease in plasma concentrations at week 52 is primarily explained by protocol-defined visit scheduling, as most QM samples at weeks 36 and 44 were collected ∼2 weeks postdosing, whereas QM samples at week 52 were collected ∼4 weeks postdosing, although weight-related changes in exposure may also have contributed. Importantly, plasma concentrations remained within a narrow range across dosing frequencies despite a wide span of body weights and dose adjustments following weight-range transitions (Supplementary Table 1), confirming the validity of the tiered dosing strategy.

Typically, patients with hemophilia with FVIII inhibitors experience greater disease burden and frequency of bleeds [46]. Despite this, no treated bleeds were reported in inhibitor-positive participants of FRONTIER3, irrespective of denecimig dosing frequency. However, the lack of bleeding in this population could be attributed to heightened parental vigilance, activity restriction in younger children [1,47], or reluctance to seek treatment with bypassing agents, owing to venous access challenges, thrombotic risk perception, or variable efficacy [17,48,49].

The bleed protection observed with denecimig in FRONTIER3 (estimated mean ABRs of 0.50 with QW [parts 1 and 2] and 0.25 with QM dosing [Part 2]) compares favorably with other recently evaluated prophylactic therapies in pediatric patients, although crosstrial comparisons should be interpreted with caution. Given that denecimig, like emicizumab, is a FVIII-mimetic bispecific antibody developed for patients with and without FVIII inhibitors, comparison with emicizumab is particularly relevant. In HAVEN 2, emicizumab prophylaxis in children aged <12 years with HA with FVIII inhibitors resulted in a model-based mean ABR of 0.3 (95% CI, 0.17-0.50) in the largest once-weekly cohort (1.5 mg/kg), while smaller cohorts receiving every-2-week (3 mg/kg) and every-4-week (6 mg/kg) dosing reported mean ABRs of 0.2 and 2.2, respectively [50]. Comparable low bleeding rates were observed with denecimig in both participants with and without FVIII inhibitors. Similar efficacy has also been reported with efanesoctocog alfa prophylaxis in children aged <12 years with severe hemophilia A without inhibitors (XTEND-Kids), in which the model-based estimated mean ABR was 0.89 (95% CI, 0.56-1.42) over 52 weeks [51].

The observed 65.7% reduction in ABR compared with prestudy FVIII prophylaxis over 26 weeks (Figure 3) demonstrated denecimig’s potent bleed prevention and potential to provide efficacy improvements over the current standard of care. Similar efficacy for denecimig QW and QM was observed in adult and adolescent patients in FRONTIER2 (65% to 95% of patients with zero bleeds over 26 weeks) [33], and in adults receiving denecimig every 2 weeks in FRONTIER4 (84% of patients with zero bleeds over 26 weeks), [52] reinforcing the durability of bleed protection with denecimig across age groups and dosing frequencies. Observed bleed rates were numerically lower over time, with estimated mean ABRs decreasing from 0.53 with denecimig QW in Part 1 to 0.42 with QW and 0.25 with QM in Part 2, which may in part reflect increasing confidence among families and caregivers that minor traumas did not always require treatment, leading to fewer reported bleeding events [53]. Baseline characteristics (including mean age and inhibitor status) were similar between those switching to QM and those remaining on QW in Part 2. Participants who switched to QM dosing (n = 31) demonstrated similar bleeding control during Part 1 to the overall QW cohort (n = 70), with an estimated mean ABR of 0.45 compared with 0.53, suggesting that switching to QM was not confined to participants with the lowest bleeding rates or demographic differences. This further highlights the favorable estimated mean ABR in the QM group of 0.25, particularly in the context of every-4-week dosing outcomes reported in A Multicenter, Open-Label, Phase III Clinical Trial to Evaluate the Efficacy, Safety, and Pharmacokinetics of Subcutaneous Administration of Emicizumab in Hemophilia A Pediatric Patients with Inhibitors (HAVEN 2) [50]. However, unmeasured factors, such as caregiver preferences toward injections, treatment burden, or thresholds for reporting minor bleeding events, may have influenced both dosing preferences and bleeding-reporting behavior [54,55]. The low ABR, high proportion of patients experiencing zero treated bleeds, and the resolution of all baseline target joints, suggests potential long-term benefits for joint health, an important goal of treatment in young children [1,6]. Target joints have been reported in young children receiving prophylaxis, highlighting that early joint damage may occur despite preventive treatment [6,56]. In FRONTIER3, all baseline target joints resolved with denecimig prophylaxis, supporting early intervention to preserve joint health and reverse joint pathology.

Beyond bleed prevention, denecimig prophylaxis resulted in measurable improvements in treatment burden and quality of life. Although FRONTIER3 employed an enhanced cartridge system rather than the final denecimig pen-injector device, [32] caregivers reported substantial reductions in the burden of treatment administration, while nearly all caregivers (98%) preferred denecimig over their child’s prior treatment. Findings from a separate device-handling study indicated that most participants (89%) preferred the denecimig pen-injector over the current emicizumab injection system, citing greater ease of use and faster preparation [32]. To place the observed improvements in health-related quality of life into context, results from the post hoc SEm analysis indicate that a substantial proportion of participants experienced PedsQL score improvements that exceeded the expected measurement variability of the instrument. Specifically, improvements greater than or equal to SEm were observed in the majority of participants for the PedsQL total score, with more than half of participants exceeding the SEm threshold across individual domains, supporting that the observed improvements were unlikely to be driven by random measurement error alone. The range of SEm values observed across PedsQL domains is consistent with values reported for the PedsQL in other paediatric populations and aligns with distribution-based methods to complement derivation of the minimal clinically important difference for the PedsQL [38]. However, because SEm-based thresholds do not incorporate patient- or caregiver-perceived change (eg, anchor-based methods), they should be interpreted as supportive rather than definitive evidence of clinical meaningfulness. Together, these findings suggest that effective bleed prevention and simplified treatment administration may translate into broader benefits for children’s daily activities and overall well-being.

The denecimig tiered dosing strategy used in FRONTIER3 aims to simplify treatment by reducing the need for frequent dose recalculations; its fixed-dose, fixed-volume approach minimizes the need to adjust doses as children grow, which may help to reduce the risk of administration errors, ease treatment burden for families, and support adherence [31]. The study design aimed to achieve an approximately equal distribution between QW and QM dosing in Part 2 across age groups. The observed distribution provides useful insights into treatment preferences and indicates confidence in the efficacy of QM dosing of denecimig, which offers potential convenience relative to established QW administration. Efficacy outcomes were consistent between regimens, and caregivers preferred both QW and QM dosing to their previous treatment. Notably, all participants chose to continue denecimig prophylaxis in FRONTIER4, reflecting sustained acceptance of both dosing schedules.

Limitations of the study include its open-label, noncomparative design and the small number of participants undergoing ITI therapy, which limits conclusions that can be drawn in this subgroup. Although the number of participants with FVIII inhibitors was substantial for a pediatric study, conclusions for this subgroup should also be interpreted with appropriate caution. In addition, approximately one-third of participants had received prior on-demand treatment, which may have influenced the observed bleeding outcomes. Future investigations could further explore the use of denecimig in patients undergoing ITI and in broader pediatric populations. An additional limitation is the reliance on patient/caregiver self-reporting of bleeding episodes, which may be subject to underreporting or overreporting.

During FRONTIER3, denecimig prophylaxis was associated with low bleeding rates, and meaningful improvements in caregiver-reported outcomes in children with HA, with or without FVIII inhibitors, with outcomes consistent across age groups and dosing regimens. Together with flexible dosing options, denecimig has the potential to redefine prophylaxis in pediatric HA. Ongoing follow-up will further define the long-term safety and durability of denecimig in the FRONTIER4 open-label extension study.

## References

[1] Srivastava A., Santagostino E., Dougall A., Kitchen S., Sutherland M., Pipe S.W. (2020). WFH Guidelines for the Management of Hemophilia, 3rd edition. Haemophilia.

[2] O’Hara J., Walsh S., Camp C., Mazza G., Carroll L., Hoxer C. (2018). The impact of severe haemophilia and the presence of target joints on health-related quality-of-life. Health Qual Life Outcomes.

[3] Monahan P.E., Baker J.R., Riske B., Soucie J.M. (2011). Physical functioning in boys with hemophilia in the U.S. Am J Prev Med.

[4] Soucie J.M., Wang C., Siddiqi A., Kulkarni R., Recht M., Konkle B.A. (2011). The longitudinal effect of body adiposity on joint mobility in young males with haemophilia A. Haemophilia.

[5] Bladen M., Main E., Hubert N., Koutoumanou E., Liesner R., Khair K. (2013). Factors affecting the Haemophilia Joint Health Score in children with severe haemophilia. Haemophilia.

[6] Manco-Johnson M.J., Abshire T.C., Shapiro A.D., Riske B., Hacker M.R., Kilcoyne R. (2007). Prophylaxis versus episodic treatment to prevent joint disease in boys with severe hemophilia. N Engl J Med.

[7] Abdelrazik N., Reda M., El-Ziny M., Rabea H. (2007). Evaluation of bone mineral density in children with hemophilia: Mansoura University children hospital (MUCH) experience, Mansoura, Egypt. Hematology.

[8] Ferri Grazzi E., Becker T., Brandt S., Duport G., Garcia Diego D.A., Lupi A. (2024). Clinical and humanistic burden of non-inhibitor haemophilia A in five European Countries: insights from the CHESS II study. Adv Ther.

[9] Gebetsberger J., Schirmer M., Wurzer W.J., Streif W. (2022). Low bone mineral density in hemophiliacs. Front Med (Lausanne).

[10] Young G. (2021). Can we do something about ICH in hemophilia?. Blood.

[11] Zwagemaker A.F., Gouw S.C., Jansen J.S., Vuong C., Coppens M., Hu Q. (2021). Incidence and mortality rates of intracranial hemorrhage in hemophilia: a systematic review and meta-analysis. Blood.

[12] Rezende S.M., Neumann I., Angchaisuksiri P., Awodu O., Boban A., Cuker A. (2024). International Society on Thrombosis and Haemostasis clinical practice guideline for treatment of congenital hemophilia A and B based on the Grading of Recommendations Assessment, Development, and Evaluation methodology. J Thromb Haemost.

[13] Teitel J. (2025). More real-world evidence on the impact of emicizumab. Res Pract Thromb Haemost.

[14] Ragni M.V. (2015). New and emerging agents for the treatment of hemophilia: focus on extended half-life recombinant clotting proteins. Drugs.

[15] Valentino L.A., Ewenstein B., Navickis R.J., Wilkes M.M. (2004). Central venous access devices in haemophilia. Haemophilia.

[16] Buckley B., Dreyfus J., Prasad M., Gayle J., Kendter J., Hall E. (2018). Burden of illness and costs among paediatric haemophilia patients with and without central venous access devices treated in US hospitals. Haemophilia.

[17] Bedoya M.A., Raffini L., Durand R., Acord M.R., Srinivasan A., Krishnamurthy G. (2020). Implantable venous access devices in children with severe hemophilia: a tertiary pediatric institutional experience. Pediatr Radiol.

[18] van den Berg H.M., Fischer K., Carcao M., Chambost H., Kenet G., Kurnik K. (2019). Timing of inhibitor development in more than 1000 previously untreated patients with severe hemophilia A. Blood.

[19] Mancuso M.E., Croteau S.E., Klamroth R. (2024). Benefits and risks of non-factor therapies: redefining haemophilia treatment goals in the era of new technologies. Haemophilia.

[20] Mahlangu J., Iorio A., Kenet G. (2022). Emicizumab state-of-the-art update. Haemophilia.

[21] Østergaard H., Lund J., Greisen P.J., Kjellev S., Henriksen A., Lorenzen N. (2021). A factor VIIIa-mimetic bispecific antibody, Mim8, ameliorates bleeding upon severe vascular challenge in hemophilia A mice. Blood.

[22] Jiménez-Yuste V. (2025). Non-factor therapies for hemophilia: achievements and perspectives. Semin Thromb Hemost.

[23] Mancuso M.E., Mahlangu J., Sidonio R., Trask P., Uguen M., Chang T. (2020). Health-related quality of life and caregiver burden of emicizumab in children with haemophilia A and factor VIII inhibitors—results from the HAVEN 2 study. Haemophilia.

[24] Alcedo Andrade P.E., Mannucci P.M., Kessler C.M. (2024). Emicizumab: the hemophilia A game-changer. Haematologica.

[25] Young G., Pipe S.W., Kenet G., Oldenburg J., Safavi M., Czirok T. (2024). Emicizumab is well tolerated and effective in people with congenital hemophilia A regardless of age, severity of disease, or inhibitor status: a scoping review. Res Pract Thromb Haemost.

[26] Kruis I.C., Driessens M.H. (2023). Pain while injecting emicizumab predominant in children, a report of Dutch patient experiences. Haemophilia.

[27] Chenuel C, Delienne S, FIDEL’HEM. PO352 therapeutic education for patients treated with Emicizumab in France. Presented at the European Association for Haemophilia and Allied Disorders (EAHAD) 2026 congress; Dublin, Ireland.

[28] Young G. (2023). The dosing conundrum of emicizumab: to waste product or not?. Res Pract Thromb Haemost.

[29] Mancuso M.E., Castaman G., Pochopien M., Aballéa S., Drzewiecka A., Hakimi Z. (2022). Cost-minimization analysis of recombinant factor VIII Fc versus emicizumab for treating patients with hemophilia A without inhibitors in Europe. J Med Econ.

[30] Lentz S.R., Chowdary P., Gil L., Lopez-Jaime F.J., Mahlangu J., Matytsina I. (2024). FRONTIER1: a partially randomized phase 2 study assessing the safety, pharmacokinetics, and pharmacodynamics of Mim8, a factor VIIIa mimetic. J Thromb Haemost.

[31] Kreilgaard M., Matytsina I., Persson P. (2025). Model-informed drug development of Mim8—a next-generation bispecific antibody for treatment of haemophilia A. Eur J Pharm Sci.

[32] Shastri L, Mulders G, Sparre T, Bellm KC, Ter-Borch G, Sidonio R (2025). Poster PO108 presented at: 18th Annual Congress of the European Association for Haemophilia and Allied Disorders (EAHAD).

[33] Mancuso M.E., Chan A.K.C., Shanmukhaiah C., Lyu C.J., Zdziarska J., Mahlangu J. (2026). Mim8 bispecific antibody prophylaxis in hemophilia A with or without inhibitors. N Engl J Med.

[34] Shankar G., Devanarayan V., Amaravadi L., Barrett Y.C., Bowsher R., Finco-Kent D. (2008). Recommendations for the validation of immunoassays used for detection of host antibodies against biotechnology products. J Pharm Biomed Anal.

[35] EMA (2017). Guideline on immunogenicity assessment of biotechnology-derived therapeutic proteins. https://www.ema.europa.eu/en/immunogenicity-assessment-biotechnology-derived-therapeutic-proteins-scientific-guideline.

[36] FDA (2019). Immunogenicity testing of therapeutic protein products—developing and validating assays for anti-drug antibody detection. https://www.fda.gov/media/119788/download.

[37] Brod M., Bushnell D.M., Busk A.K., Neergaard J.S. (2025). Development and validation of the Child Hemophilia Treatment Experience Measure: a new observer-reported outcome measure. Haemophilia.

[38] Varni J.W., Seid M., Kurtin P.S. (2001). PedsQL 4.0: reliability and validity of the Pediatric Quality of Life Inventory version 4.0 generic core scales in healthy and patient populations. Med Care.

[39] EMA (2018). Guideline on the clinical investigation of recombinant and human plasma-derived factor VIII products. https://www.ema.europa.eu/en/documents/scientific-guideline/guideline-clinical-investigation-recombinant-and-human-plasma-derived-factor-viii-products-revision-2_en.pdf.

[40] (2013). World Medical Association Declaration of Helsinki: ethical principles for medical research involving human subjects. JAMA.

[41] (2001). ICH harmonized tripartite guideline: guideline for good clinical practice. J Postgrad Med.

[42] St Clair-Jones A., Prignano F., Goncalves J., Paul M., Sewerin P. (2020). Understanding and minimising injection-site pain following subcutaneous administration of biologics: a narrative review. Rheumatol Ther.

[43] Usach I., Martinez R., Festini T., Peris J.E. (2019). Subcutaneous injection of drugs: literature review of factors influencing pain sensation at the injection site. Adv Ther.

[44] Zijlstra E., Jahnke J., Fischer A., Kapitza C., Forst T. (2018). Impact of injection speed, volume, and site on pain sensation. J Diabetes Sci Technol.

[45] Lund J., Jensen K., Burnier L., Ezban M. (2023). In vitro effects of combining Mim8 with factor VIII, FVIIa, and activated prothrombin complex concentrates in thrombin generation assays. J Thromb Haemost.

[46] Oladapo A.O., Lu M., Walsh S., O’Hara J., Kauf T.L. (2018). Inhibitor clinical burden of disease: a comparative analysis of the CHESS data. Orphanet J Rare Dis.

[47] Bérubé S., Ogez D., Aramideh J., Amesse C., Bourque C.J., Longpré C. (2021). It’s difficult to say no: development of a parenting booklet about physical activity restrictions and recommendations in pediatric hemophilia. Health Psychol Open.

[48] Coppola A., Franchini M., Castaman G., Santagostino E., Santoro C., Santoro R.C. (2018). Treatment regimens with bypassing agents in patients with hemophilia a and inhibitors: a survey from the Italian Association of Hemophilia Centers (AICE). Semin Thromb Hemost.

[49] Santagostino E., Mancuso M.E., Rocino A., Mancuso G., Scaraggi F., Mannucci P.M. (2006). A prospective randomized trial of high and standard dosages of recombinant factor VIIa for treatment of hemarthroses in hemophiliacs with inhibitors. J Thromb Haemost.

[50] Young G., Liesner R., Chang T., Sidonio R., Oldenburg J., Jiménez-Yuste V. (2019). A multicenter, open-label phase 3 study of emicizumab prophylaxis in children with hemophilia A with inhibitors. Blood.

[51] Malec L., Peyvandi F., Chan A.K.C., Königs C., Zulfikar B., Yuan H. (2024). Efanesoctocog alfa prophylaxis for children with severe hemophilia A. N Engl J Med.

[52] Matsushita T, Banchev A, Bovet J, Chowdary P, García Fernández LM, Jiménez-Yuste V (2026). Denecimig (Mim8) prophylaxis once-every-2-weeks for hemophilia A with or without inhibitors: 26-week results from FRONTIER. Blood Adv.

[53] Callaghan M.U., Asikanius E., Lehle M., Oldenburg J., Mahlangu J., Uguen M. (2022). Untreated bleeds in people with hemophilia A in a noninterventional study and intrapatient comparison after initiating emicizumab in HAVEN 1-3. Res Pract Thromb Haemost.

[54] Garcia V.C., Mansfield C., Pierce A., Leach C., Smith J.C., Afonso M. (2024). Patient and caregiver preferences for haemophilia treatments: a discrete-choice experiment. Haemophilia.

[55] Benton M., Patel A.M., Shi L., Monnette A., Hong D., Kruse-Jarres R. (2021). Assessing patient and caregiver preferences for treatment of haemophilia A: a discrete choice experiment. Haemophilia.

[56] Mullins E.S., Stasyshyn O., Alvarez-Román M.T., Osman D., Liesner R., Engl W. (2017). Extended half-life pegylated, full-length recombinant factor VIII for prophylaxis in children with severe haemophilia A. Haemophilia.

