# Supplementary material for: Denecimig (Mim8) prophylaxis in children with hemophilia A with or without factor VIII inhibitors
Source: Res Pract Thromb Haemost. 2026 Aug 20;10(6):106914. doi: 10.1016/j.rpth.2026.106914 (PMC13635261; doi:10.1016/j.rpth.2026.106914)
Supplement: Supplementary file 1 [file mmc1.docx]

**Denecimig (Mim8) Prophylaxis in Children With Haemophilia A With or Without Factor VIII Inhibitors: Supplementary material**

Table of contents

[FRONTIER3 investigators:	2](#_Toc236306585)

[Supplementary Methods:	2](#_Toc236306586)

[Supplementary Table 1. Denecimig QW and QM prophylaxis dosing	3](#_Toc236306587)

[Supplementary Table 2. Race distribution of study participants	4](#_Toc236306588)

[Supplementary Table 4. Most frequent TEAEs by system organ class and preferred term	7](#_Toc236306589)

[Supplementary Table 5. *Post hoc* analysis for descriptive statistics and SEm of PedsQL change from baseline to end of Part 2	10](#_Toc236306590)

[Supplementary Figure 1. Participant flow	11](#_Toc236306591)

[Supplementary Figure 2. Anti-denecimig antibodies over time in relation to denecimig concentration	12](#_Toc236306592)

[Supplementary Figure 3. Laboratory parameters	14](#_Toc236306593)

[Supplementary Figure 4. Change in PedsQL subdomain scores with denecimig prophylaxis from baseline to week 52 in participants aged ≥5 years	16](#_Toc236306594)

**FRONTIER3 investigators:**

Sanjay Ahuja, Jayanthi Alamelu, Nataly Apollonsky, Shashikant Apte, Rubén Berrueco, Manuel Carcao, M. Elaine Eyster, C.J. Fijnvandraat, Kapil Garg, Deepan Gosrani, Naoaki Hori, Irena Woznica Karczmarz, Gili Kenet, Paula Kjöllerström, Elzbieta Latos-Grazynska, Matteo Luciani, Chuhl Joo Lyu, Johnny Mahlangu, Nita Radhakrishnan, Freimut Schilling, Janice Staber, Jing Sun, Runhui Wu, Renchi Yang, Yung-Li Yang, Chur Woo You, Min Zhou

**Supplementary Methods:**

Anti-denecimig antibody assay

Plasma samples were assessed for the presence of anti-denecimig antibodies using validated electrochemiluminescence (ECL) assays in a tiered approach. All samples were initially screened for anti-denecimig antibodies, and samples above the screening cut point were analysed in the confirmatory assay. The level of anti-denecimig antibodies was determined by titration of samples confirmed positive for anti-denecimig antibodies.

The assay was a bridging ECL assay with an acid dissociation step (glycine–hydrogen chloride buffer, 300 mM, pH 2.5) to dissociate the binding between anti-denecimig antibodies and potentially interfering denecimig. Acid treatment was followed by neutralisation with tris(hydroxymethyl)aminomethane (TRIS) buffer (1 M, pH 9.5) and incubation with both biotinylated denecimig and Sulfo-Tag-labelled denecimig to bind any anti-denecimig antibodies. The samples were transferred to an MSD streptavidin-coated plate to immobilise the anti-denecimig antibody:denecimig complexes. Substrate (MSD Read Buffer) was added, the plate was loaded into an MSD instrument, and voltage was applied to the plate electrodes, causing light emission from the Sulfo-Tag label, which was recorded. To confirm the presence of anti-denecimig antibodies, samples containing an excess of unlabelled denecimig were analysed in parallel with the standard samples. The presence of denecimig-specific antibodies was confirmed if the response in the sample containing excess unlabelled denecimig was inhibited compared with the standard sample. The assay had a sensitivity of <1 ng/mL of anti-denecimig antibody positive control in the absence of drug and could tolerate 35 μg/mL denecimig. Inter-assay and intra-assay signal variability (coefficient of variation) were <15%.

**Supplementary Table 1. Denecimig QW and QM prophylaxis dosing**

| **Weight band (kg)** | **Dose QW** | | | | **Dose QM** | |
| --- | --- | --- | --- | --- | --- | --- |
|  | Loading dose | | Maintenance dose | | Maintenance dose | |
|  | Total dose (mg) | Dose per injection (mg) | Total dose (mg) | Dose per injection (mg) | Total dose (mg) | Dose per injection (mg) |
| <15 | 9.0 | 9.0 | 1.6 | 1.6 | 9.0 | 9.0 |
| 15–<45* | 24.0 | 4.0 | 4.0 | 4.0 | 20.0 | 20.0 |
|  |  | 20.0 |  |  |  |  |
| ≥45 | 55.0 | 9.0 | 9.0 | 9.0 | 46.0 | 46.0 |
|  |  | 46.0 |  |  |  |  |

**Three participants in FRONTIER3 crossed the 15 kg body-weight threshold during the study and therefore transitioned from the <15 kg to the 15–<45 kg weight band according to the protocol-defined dosing scheme. Two participants receiving QM dosing had their maintenance dose adjusted from 9 mg to 20 mg. One additional participant temporarily fluctuated around the 15 kg threshold between visits 4 and 5, resulting in the administration of a 1.6 mg dose at visit 4, before returning to a 4 mg dose at visit 5. These dose adjustments were protocol-consistent and were not associated with bleeding episodes or treatment-emergent adverse events.*

*QM, once-every-month; QW, once-every-week.*

**Supplementary Table 2. Race distribution of study participants**

|  | **Aged 1–5 years** | | | | **Aged 6–11 years** | | | | **Overall  total** |
| --- | --- | --- | --- | --- | --- | --- | --- | --- | --- |
|  | **PPX** | **OD** | **ITI/ PUP** | **Total** | **PPX** | **OD** | **ITI/ PUP** | **Total** |  |
| **Number of participants, n** | 18 | 15 | 3 | 36 | 22 | 10 | 2 | 34 | 70 |
| **Race, n (%)** |  |  |  |  |  |  |  |  |  |
| Asian | 4 (22.2) | 9 (60.0) | 2 (66.7) | 15 (41.7) | 10 (45.5) | 7 (70.0) | 2 (100) | 19 (55.9) | 34  (48.6) |
| Black or African American | 0 | 4 (26.7) | 0 | 4 (11.1) | 2  (9.1) | 1 (10.0) | 0 | 3 (8.8) | 7  (10.0) |
| White | 14 (77.8) | 1  (6.7) | 1 (33.3) | 16 (44.4) | 9 (40.9) | 1 (10.0) | 0 | 10 (29.4) | 26  (37.1) |
| Other | 0 | 1  (6.7) | 0 | 1  (2.8) | 1  (4.5) | 1 (10.0) | 0 | 2  (5.9) | 3  (4.3) |

*ITI, immune tolerance induction; n, number of patients; OD, on-demand; PPX, prophylaxis; PUP, previously untreated patient.*

**Supplementary Table 3. Summary of treatment history**

|  | **1−5 years** | **6−11 years** | **Total** |
| --- | --- | --- | --- |
| **Number of participants** | **36** | **34** | **70** |
| **Regimen before entering the trial, N (%)** |  |  |  |
| **Prophylaxis** | **21 (58.3)** | **23 (67.6)** | **44 (62.9)** |
| **On demand** | **15 (41.7)** | **13 (38.2)** | **28 (40.0)** |
| **Time on prophylaxis (days)*** |  |  |  |
| **N** | **21** | **23** | **44** |
| **Mean (SD)** | **355 (34)** | **347 (68)** | **351 (54)** |
| **Median** | **366** | **366** | **366** |
| **Dosing frequencies on prophylaxis, N (%)** |  |  |  |
| **N** | **21 (100)** | **23 (100)** | **44 (100)** |
| **QD** | **-** | **1 (4.3)** | **1 (2.3)** |
| **QOD** | **3 (14.3)** | **2 (8.7)** | **5 (11.4)** |
| **Q3D** | **1 (4.8)** | **1 (4.3)** | **2 (4.5)** |
| **Q4D** | **-** | **1 (4.3)** | **1 (2.3)** |
| **Once per week** | **1 (4.8)** | **-** | **1 (2.3)** |
| **Twice per week** | **13 (61.9)** | **15 (65.2)** | **28 (63.6)** |
| **Three times per week** | **8 (38.1)** | **8 (34.8)** | **16 (36.4)** |
| **Number of treated bleeds on prophylaxis** | **39** | **55** | **94** |

****If a participant has been on multiple prophylaxis regimens or on demand treatments then the time on prophylaxis or on demand includes the time on all regimens respectively, and the same goes for number of bleeds. N, number of participants; QD, once daily; QOD, every 2 days; Q3D, every 3 days; Q4D, every 4 days; SD, standard deviation.***

**Supplementary Table 4. Most frequent TEAEs by system organ class and preferred term**

| **Most frequent TEAEs by system organ class / preferred term** | **Denecimig QW**  **(Parts 1 and 2)** | | **Denecimig QM**  **(Part 2)** | | **Total** | |
| --- | --- | --- | --- | --- | --- | --- |
|  | **N (%)** | **E [R]** | **N (%)** | **E [R]** | **N (%)** | **E [R]** |
| **Number of participants, N** | 70 | - | 32 | - | 70 | - |
| Infections and infestations* | 31 (44.3) | 57 [1.04] | 13 (40.6) | 18 [1.12] | 38  (54.3) | 75 [1.06] |
| Upper respiratory tract infection | 14 (20.0) | 15 [0.27] | 1 (3.1) | 1 [0.06] | 15 (21.4) | 16 [0.23] |
| Nasopharyngitis | 5 (7.1) | 7 [0.13] | 2 (6.3) | 3 [0.19] | 6 (8.6) | 10 [0.14] |
| Bronchitis | 4 (5.7) | 4 [0.07] | - | - | 4 (5.7) | 4 [0.06] |
| General disorders and administration site conditions | 20  (28.6) | 41  [0.75] | 1 (3.1) | 5  [0.31] | 20  (28.6) | 46  [0.65] |
| Injection site reaction | 7 (10.0) | 26 [0.48] | 1 (3.1) | 5 [0.31] | 7 (10.0) | 31 [0.44] |
| Pyrexia | 14 (20.0) | 15 [0.27] | - | - | 14 (20.0) | 15 [0.21] |
| Injury, poisoning and procedural complications | 13  (18.6) | 30 [0.55] | 3  (9.4) | 3  [0.19] | 16  (22.9) | 33  [0.47] |
| Incorrect dose administered | 2 (2.9) | 6 [0.11] | - | - | 2 (2.9) | 6 [0.08] |
| Fall | 3 (4.3) | 4 [0.07] | 1 (3.1) | 1 [0.06] | 4 (5.7) | 5 [0.07] |
| Product communication issue | 3 (4.3) | 3 [0.05] | - | - | 3 (4.3) | 3 [0.04] |
| Investigations | 10 (14.3) | 15 [0.27] | 5  (15.6) | 9  [0.56] | 13  (18.6) | 24  [0.34] |
| Prothrombin fragment 1.2 increased | 7 (10.0) | 7 [0.13] | 4 (12.5) | 4 [0.25] | 9 (12.9) | 11 [0.16] |
| Blood alkaline phosphatase increased | 2 (2.9) | 3 [0.05] | - | - | 2 (2.9) | 3 [0.04] |
| Fibrin D-dimer increased | 1 (1.4) | 1 [0.02] | 1 (3.1) | 1 [0.06] | 2 (2.9) | 2 [0.03] |
| Gastrointestinal disorders | 9 (12.9) | 10  [0.18] | 1  (3.1) | 2  [0.12] | 9  (12.9) | 12  [0.17] |
| Vomiting | 3 (4.3) | 3 [0.05] | - | - | 3 (4.3) | 3 [0.04] |
| Dental caries | 2 (2.9) | 2 [0.04] | - | - | 2 (2.9) | 2 [0.03] |
| Diarrhoea | 1 (1.4) | 1 [0.02] | 1 (3.1) | 1 [0.06] | 2 (2.9) | 2 [0.03] |
| Respiratory, thoracic and mediastinal disorders | 9  (12.9) | 10  [0.18] | 2  (6.3) | 2  [0.12] | 10  (14.3) | 12  [0.17] |
| Cough | 8 (11.4) | 8 [0.15] | 1 (3.1) | 1 [0.06] | 9 (12.9) | 9 [0.13] |
| Nasal congestion | 1 (1.4) | 1 [0.02] | - | - | 1 (1.4) | 1 [0.01] |
| Rhinitis allergic | 1 (1.4) | 1 [0.02] | - | - | 1 (1.4) | 1 [0.01] |
| Musculoskeletal and connective tissue disorders | 4 (5.7) | 8  [0.15] | 1 (3.1) | 2  [0.12] | 5  (7.1) | 10  [0.14] |
| Joint effusion | 1 (1.4) | 3 [0.05] | - | - | 1 (1.4) | 3 [0.04] |
| Arthralgia | 1 (1.4) | 1 [0.02] | 1 (3.1) | 1 [0.06] | 2 (2.9) | 2 [0.03] |
| Myalgia | 1 (1.4) | 1 [0.02] | 1 (3.1) | 1 [0.06] | 2 (2.9) | 2 [0.03] |
| Skin and subcutaneous tissue disorders | 5  (7.1) | 6  [0.11] | 1  (3.1) | 1  [0.06] | 6  (8.6) | 7 [0.10] |
| Dermatitis contact | 2 (2.9) | 2 [0.04] | - | - | 2 (2.9) | 2 [0.03] |
| Dry skin | 1 (1.4) | 1 [0.02] | - | - | 1 (1.4) | 1 [0.01] |
| Eczema | 1 (1.4) | 1 [0.02] | - | - | 1 (1.4) | 1 [0.01] |

*Within each system organ class, only the 3 most frequently reported preferred terms (based on total number of events) are presented. *No infestations were reported.
E, number of adverse events; N, number of patients; QM, once-every-month; QW, once-every-week; R, number of adverse events per patient year of exposure (E/total exposure time in study); TEAE, treatment emergent adverse event.*

**Supplementary Table 5. *Post hoc* analysis for descriptive statistics and SEm of PedsQL change from baseline to end of Part 2**

| **Domain** | **Number of items** | **Number of observations** | **Mean** | **SD** | **SEm** | **Proportion of patients with score ≥SEm* (%)** |
| --- | --- | --- | --- | --- | --- | --- |
| **Total score** | 23 | 31 | 13.6 | 18.4 | 4.59 | 67.74 |
| Physical functioning | 8 | 31 | 10.4 | 22.6 | 7.04 | 51.61 |
| Emotional functioning | 5 | 31 | 13.9 | 16.3 | 7.74 | 70.97 |
| Social functioning | 5 | 31 | 15.6 | 21.7 | 9.04 | 61.29 |
| School functioning | 5 | 31 | 16.6 | 24.2 | 10.41 | 51.61 |

**Refers to the percentage of patients with an absolute change in the score higher or equal to the SEm threshold. Includes participants who have reached the end of Part 2, and who have responded to the questionnaire at baseline and at week 52. SEm calculated as the product of the SD of the total (or respective domain score) by the square root of 1-alpha, in which alpha is the Cronbach alpha reliability coefficient. In all cases, the variables analysed refer to the absolute change in scores between baseline (week 0) and the end of treatment (week 52).*

*PedsQL, Pediatric Quality of Life Inventory; SD, standard deviation; SEm, standard error of measurement.*

**Supplementary Figure 1. Participant flow**

*The run-in was mandatory for previously treated participants with prophylaxis treatment, with the exception of Chinese participants, for whom a run-in period was not required according to country-specific protocol requirements; 36 (51%) entered the run-in for a minimum of 26 weeks. One participant did not meet eligibility criteria at screening, and one withdrew during the run-in period because the participant’s parents declined treatment with an investigational product. All enrolled patients (n=70) completed both study parts, with no withdrawals or treatment discontinuations.*

*QM, once-every-month; QW, once-every-week.*

**Supplementary Figure 2. Anti-denecimig antibodies over time in relation to denecimig concentration**

*A red dot on the plot indicates a positive ADA sample. If no positive ADA sample was taken at the exact time of the denecimig concentration measurement, no red dots appear for that participant, even if they were ADA positive at some point during the study. However, they will still appear as an ADA positive participant, indicated by a blue line. A blue dot indicates a negative ADA sample for an ADA positive participant.*

*ADA, anti-denecimig antibody.*

**Supplementary Figure 3. Laboratory parameters**

Panel 3a

Panel 3b

*a) Individual D-dimer profiles b) Individual prothrombin fragment 1 and 2 profiles. For both panels, horizontal black lines refer to lower and upper range limits, and PPX, OD, ITI and PUP refer to the previous treatment before starting on denecimig.*

*Several outlier values are not shown in the figure. For D-dimer, excluded values were observed in 9 patients: day 0 (1,003 µg/L DDU); days 56 and 252 (1,818 and 2,272 µg/L DDU); days 56 and 365 (1,040 and 1,149 µg/L DDU); day 0 (14,,526 µg/L DDU); day 182 (2262 µg/L DDU); day 308 (2,226 µg/L DDU); day 182 (1,289 µg/L DDU); days 56 and 308 (3,497 and 4,458 µg/L DDU); and day 0 (8,984 µg/L DDU).*

*For prothrombin fragment 1+2, excluded values were observed in 10 patients: day 84 (12,000 pmol/L); day 56 (12,000 pmol/L); days 28, 56 and 182 (12,000, 2,920 and 1,200 pmol/L); day 252 (9,260 pmol/L); day 329 (5,640 pmol/L); Day 252 (11,570 pmol/L); day 182 (5,400 pmol/L); day 182 (5,887 pmol/L); day 56 (3,250 pmol/L); and day 364 (4,157 pmol/L).*

*ITI, immune tolerance induction; OD, on-demand; PPX, prophylaxis; PUP, previously untreated patient; QW, once-every-week.*

**Supplementary Figure 4. Change in PedsQL subdomain scores with denecimig prophylaxis from baseline to week 52 in participants aged ≥5 years**

*Error bars indicate SD. Grey diamonds indicate baseline domain scores. Blue diamonds represent end of Part 2 mean domain scores for the overall population, QW, and QM dosing groups. A higher score indicates better physical functioning. Results from the PedsQL questionnaires are obtained from the Parent Report for Children (aged 8–12 years) v4.0 and the Parent Report for Young Children (aged 5–7 years) v4.0; data from these two versions were pooled. Scores range from 0 to 100. Mean change and SD include participants who reached end of Part 2 (week 52), and who had responded to the questionnaire at baseline and week 52.
PedsQL, Pediatric Quality of Life Inventory; PRO, patient-reported outcome; QM, once-every-month; QW, once-every-week; SD, standard deviation.*
